# Characterization of METTL3/14-mediated m^6^A modification in human transcriptome using Nanopore direct RNA sequencing

**DOI:** 10.1371/journal.pgen.1012278

**Published:** 2026-09-01

**Authors:** Emily Kurtyan, Andrew J. Stein, Kelly J. Abdalla, Zhangerjiao Yuan, Miten Jain, Fadia Ibrahim

**Affiliations:** 1 Department of Biochemistry and Molecular Biology, Sidney Kimmel Medical College, Thomas Jefferson University, Philadelphia, Pennsylvania, United States of America; 2 Department of Bioengineering, Northeastern University, Boston, Massachusetts, United States of America; 3 Department of Physics, Northeastern University, Boston, Massachusetts, United States of America; 4 Khoury College of Computer Sciences, Northeastern University, Boston, Massachusetts, United States of America; Olivia Newton-John Cancer Research Institute, AUSTRALIA

## Abstract

Post-transcriptional RNA modifications modulate diverse aspects of RNA metabolism. N^6^-methyladenosine (m^6^A), one of the most abundant internal RNA modifications, is deposited by the core methyltransferase complex, METTL3 and METTL14. Oxford Nanopore Technologies (ONT) platform permits direct, single RNA molecule sequencing while preserving native modifications. However, without rigorous benchmarking, the accuracy and reproducibility of modification detection remain uncertain. Here, we leveraged ONT to comprehensively profile bona fide m^6^A modifications in cellular RNAs at single-nucleotide resolution by integrating two direct RNA sequencing chemistries (RNA002 and RNA004) with the m6Anet and Dorado modification-detection models. We independently depleted METTL3 and METTL14 in human cells and rigorously validated modification calls through several assays and independent orthogonal methods (GLORI and miCLIP). We find that Dorado detected a higher number of m^6^A events and enabled simultaneous detection of other RNA modifications (5-methylcytosine, pseudouridine, and inosine). Pairing Dorado with an *in vitro* transcribed, unmodified control under stringent filtering, we provide compelling evidence supporting a global reduction in m^6^A sites and stoichiometry within coding sequences and across genes, particularly in highly modified genes and sites, and at consensus DRACH motifs. We report a differential and complex regulation of modified transcripts, accompanied by a global reduction in poly(A) tail length. Notably, METTL3 and METTL14 depletion produced distinct transcript-specific effects, supporting non-redundant roles within the m^6^A writer complex. Together, our study illustrates a notable advancement of ONT capabilities and establishes a robust transcriptome-wide framework for RNA modification detection, thereby laying the groundwork for exploring the contribution of METTL3/METTL14 to cellular functions and disease.

## Author summary

N^6^-methyladenosine (m^6^A) modifications regulate RNA metabolism by dynamically modulating RNA processing, translation, stability, and decay. This modification is primarily deposited by the METTL3/METTL14 complex, facilitating various cellular processes. Oxford Nanopore Technologies enables direct RNA sequencing (dRNA-Seq) at single-molecule resolution without disrupting its associated modifications. However, despite continued advancements, several challenges remain unresolved. To harness the full potential of this technology and uncover key biological insights into m^6^A modifications, we evaluated m6Anet and Dorado modification detection models and the two existing dRNA-Seq chemistries (RNA002/RNA004) following METTL3 or METTL14 depletion in human cells. We report genome- and transcriptome-wide changes in m^6^A sites and stoichiometry, as well as a global reduction of m^6^A levels across highly modified genes/sites, coupled with poly(A) tail shortening following METTL3/14 depletion. We reveal a functional complexity of METTL3/14-mediated regulation of modified transcripts and lay the foundation for understanding how post-transcriptional RNA modifications govern mRNA fate and contribute to disease pathogenesis.

## Introduction

Messenger RNAs (mRNAs) are altered with diverse chemical modifications that modulate several aspects of their lifecycle, including pre-mRNA splicing, export, localization, translation, and decay [1–3]. N^6^-methyladenosine (m^6^A) is the most prevalent modification in eukaryotic mRNAs and one of the most recognized epitranscriptomic regulators of gene expression. This modification influences a variety of physiological processes, including differentiation, embryonic development, and cellular stress responses [4–7]. m^6^A is deposited by a heterodimeric methyltransferase core complex comprising METTL3 and METTL14, supported by accessory proteins [8,9]. METTL3 plays a catalytic role, while METTL14 facilitates RNA binding. METTL3 methylates the N^6^ position of adenosine in a consensus DRACH (D = A/G/U; R = A/G; and H = A/C/U) sequence [10,11]. m^6^A deposition is a dynamic and reversible process in the cell, involving a cycle of methylation and demethylation. The m^6^A modification is recognized by reader proteins, including YTHDF1/2/3 and YTHDC1/2, and can be removed by eraser enzymes FTO and ALKBH5 [12–16]. A prominent function of m^6^A is mRNA decay, in which YTHDF2 recognizes m^6^A and recruits the CCR4-NOT complex, which in turn deadenylates mRNA and initiates degradation [13]. Conversely, m^6^A within RNA transcripts can also promote transcript stability through the inhibition of deadenylation [17] or through recognition by IGF2 BP proteins [18]. However, the context-dependent mechanisms that determine whether m^6^A promotes RNA decay or stabilization remain incompletely understood.

The advent of next-generation sequencing (NGS) technologies has rapidly advanced the profiling of the transcriptome and epitranscriptome, revealing many aspects of RNA function and metabolism. Despite the advantages of NGS methods, existing technologies including short-read sequencing suffer from several limitations, among which is the lack of isoform-level resolution and sensitivity in modification detection due to reliance on cDNA generation and short sequencing reads. Classical NGS-based m^6^A mapping methods involve antibody-based techniques, including methylated RNA immunoprecipitation sequencing (MeRIP-Seq) [10] and methylation individual-nucleotide-resolution crosslinking and immunoprecipitation (miCLIP) [19], as well as antibody-independent approaches, including MazF-based transcriptome-endonuclease RNA sequencing (MAZTER-Seq) [20] and deamination adjacent to RNA modification targets sequencing (DART-Seq) [21]. While these methods have provided unprecedented insights into the function and regulation of m^6^A in cellular RNAs, they also have limitations including high false-positive rates from non-specific antibody binding, inaccurate estimations of modification stoichiometry, insufficient resolution to precisely identify modified sites, and restriction to specific sequence contexts. Importantly, analyses involving short-read sequencing are often aggregated to the gene level, which cannot detect molecular events occurring at the single RNA-molecule level. Direct correlation of RNA modifications and RNA features is also limited.

Oxford Nanopore Technologies (ONT) presents a unique platform that allows direct long-read sequencing of native RNA molecules, preserving RNA modifications, including m^6^A, as well as the poly(A) tail. In this technology, single RNA molecules are threaded through membrane-bound nanopores that detect changes in ionic current as RNA molecules pass through the pore. Nucleotide bases and their modifications are identified via deconvolution of these electrical signals using deep learning algorithms [22]. The standard nanopore-based library generation protocol involves selection of polyadenylated RNA molecules followed by sequential ligation of DNA adapters to the 3′ ends of RNAs to enable retention of polyadenylated RNAs. The addition of a sequencing adapter that is equipped with a motor protein allows the initiation of RNA sequencing [23]. Compared to unmodified bases, RNA modifications shift the current intensities; these shifts are used to computationally identify modified bases. For instance, the m^6^A modification can be detected by approaches that rely on basecalling errors (Epinano [24] and Nanom6A [25]), training data from synthetic sequences (m6Anet [26]), or statistical testing of raw signals between two conditions (xPore [27]).

Recently, ONT updated its direct RNA sequencing (dRNA-Seq) chemistry from the SQK-RNA002 to the SQK-RNA004 chemistry to increase sequencing throughput and accuracy. The RNA004 chemistry incorporates a new flow cell, a faster motor protein, and an improved basecalling model, Dorado. Dorado can simultaneously call multiple modifications, including m^6^A, 5-methylcytosine (m^5^C), pseudouridine (Ψ), and inosine. This capability becomes particularly powerful when examining the functional roles of the m^6^A writer complex components METTL3 and METTL14 in regulating cellular RNAs. Despite this advancement and its rapid adoption by the community, several key challenges remain unresolved. Recent benchmarking studies of various models have revealed that current detection tools tend to produce high false-positive rates and underestimate m^6^A stoichiometry [28–32]. Evaluation of the performance and accuracy of existing models has largely relied on synthetic substrates with limited biological resources. Here, we perform ONT dRNA-Seq using our established True End-to-end RNA Sequencing (TERA-Seq) method [33,34] to robustly evaluate and profile m^6^A methylation in HeLa cells using both ONT dRNA-Seq chemistries (SQK-RNA002 and -RNA004) and compatible m^6^A calling models (m6Anet and Dorado). Using an *in vitro* transcribed (IVT) unmodified control, we showcase the first comparison between METTL3 and METTL14 by evaluating the m^6^A calling performance of two models using datasets generated from HeLa cells following METTL3 or METTL14 depletion. We examine how depletion of METTL3 and METTL14 influences global m^6^A levels, abundance of mRNAs, and poly(A) tail lengths. Our global assessments reveal patterns consistent with the specificity of METTL3/14 depletion, including reduction of m^6^A modification levels in consensus DRACH motifs, reduction of the total number of m^6^A methylation sites, and reduction of m^6^A modification levels across genes categorized based on their modification ratios. We further report trending differential regulation of modified transcripts, enrichment of specific pathways, distinct and overlapping roles of METTL3/14, and a global reduction of poly(A) tail length after METTL3/METTL14 depletion. Together, our study reveals the complexity of METTL3/14-mediated regulation of m^6^A-modified transcripts and provides a rich resource for understanding how METTL3/14-mediated m^6^A modification impacts gene expression and cellular functions.

## Results

### Nanopore dRNA-Seq of HeLa transcriptome in METTL3/14-depleted cells

**Fig 1:**
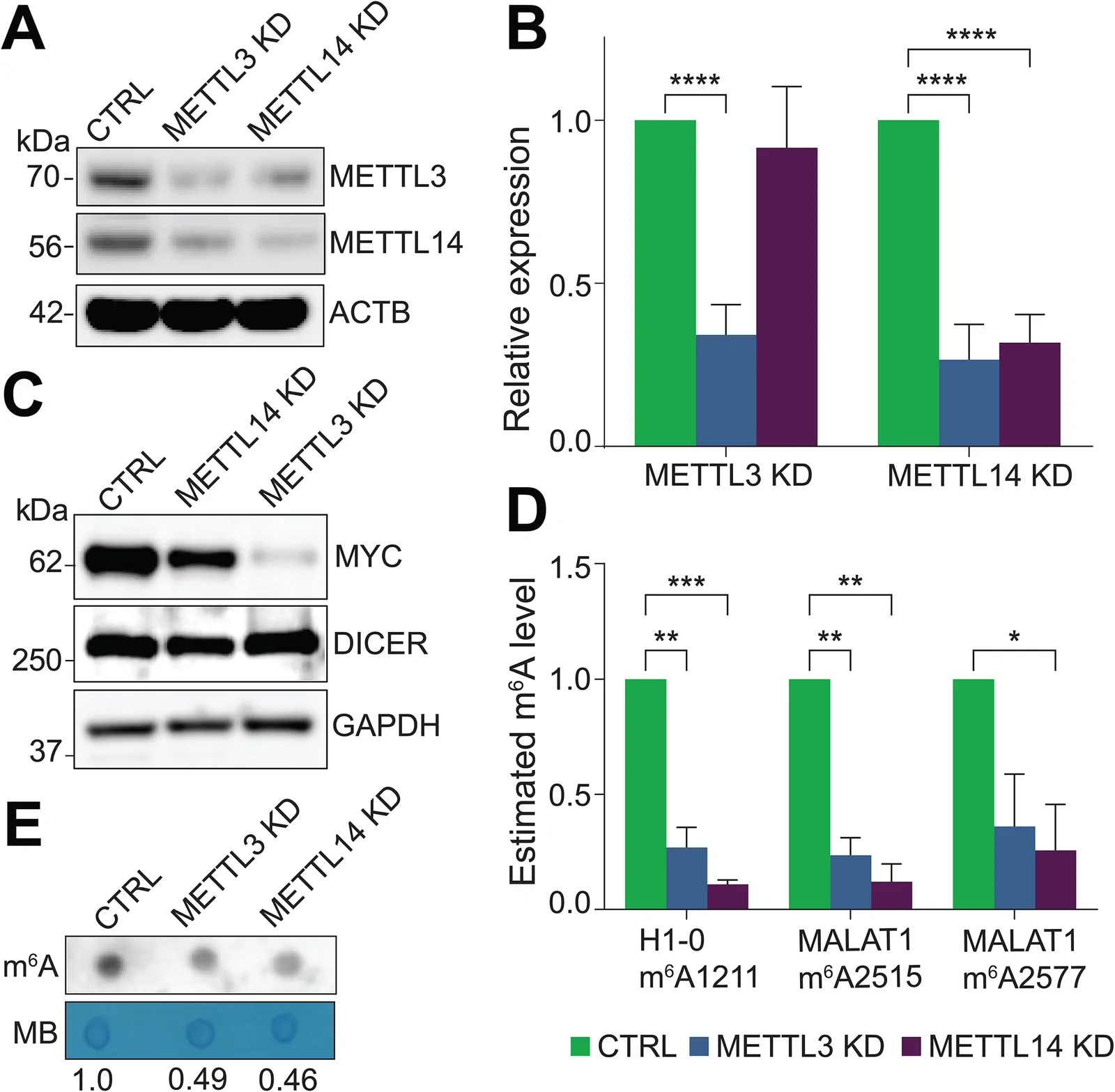
Knockdown and validation of METTL3 and METTL14 in HeLa cells. (**A**) Representative images of western blot following control (CTRL) siRNA or METTL3 and METTL14 siRNA-mediated knockdown (KD) with the corresponding antibodies. β-actin (ACTB) was used as a loading control. (**B**) Relative mRNA expression levels of METTL3 and METTL14 quantified by qPCR in CTRL and METTL3/14 KD performed using three technical replicates. Normalized Ct values (ΔCt) relative to GAPDH expression are shown as mean ± standard deviation. Statistical significance was assessed using two-way ANOVA with Dunnett’s multiple-comparison tests (****p < 0.0001). (**C**) Representative images of western blot following METTL3 or METTL14 siRNA KD with the corresponding antibodies. GAPDH was used as a loading control. (**D**) SELECT assay quantification of site-specific m^6^A modification levels on selected transcripts in control (CTRL), METTL3, and METTL14 knockdown (KD) cells, shown as relative modification levels from three technical replicates. Normalized Ct values (ΔCt) to transcript expression are displayed as mean ± standard deviation of the mean. *p < 0.05, **p < 0.01, and ***p < 0.001 with two-way ANOVA with Dunnett’s multiple comparisons test performed on normalized Ct values. (**E**) Representative images of m^6^A dot blot following METTL3 or METTL14 KD. MB, methylene blue represents loading control of RNA samples. m^6^A, N^6^-methyladensoine.

The RNA002 chemistry has been extensively adopted since the inception of Nanopore dRNA-Seq, making it a well-documented reference for benchmarking and comparison across studies. Thus, to assess the ability of Nanopore dRNA-Seq to detect m^6^A modification levels in the human transcriptome, we first attempted to use the RNA002 chemistry as a baseline to evaluate m^6^A modification levels in METTL3/METTL14-depleted HeLa cells. We isolated total RNAs and cytoplasmic extracts from cells transfected with scramble siRNA control (CTRL), and from cells separately depleted of METTL3 (METTL3 KD) and METTL14 (METTL14 KD) with siRNAs. We detected a substantial depletion of both methyltransferase enzymes in their respective KD cells compared to CTRL cells both at the protein and RNA levels (~75–90%) (Fig 1A and 1B and S1 Table). To experimentally validate the depletion of METTL3/METTL14, we selected a known METTL3-dependent m^6^A substrate, MYC [35]. We obtained a significant reduction of MYC protein upon depletion of METTL3 (~90%) and METTL14 (~70%) without any impact on the protein level of the METTL3-independent m^6^A substrate, DICER1 (Fig 1C), reproducing results consistent with previously reported observations [35,36]. Utilizing SELECT [37], a method that relies on the ability of m^6^A to hinder the single-base elongation activity of DNA polymerases and the nick ligation efficiency of ligases, we evaluated m^6^A deposition on known m^6^A-modified RNAs, including H1-0 mRNA and MALAT1 long non-coding RNA, using unmodified adenosine sites in each RNA as controls (S1 Table). We targeted one m^6^A site in H1-0 (m^6^A; 1211) and two sites in MALAT1 (m^6^A; 2515 and 2577) and observed a significant reduction of all examined m^6^A sites in KD cells compared to CTRL (Fig 1D). To evaluate the consequences of METTL3 and METTL14 depletion on global m^6^A levels, we performed an m^6^A dot blot assay and detected about ~50% global reduction in m^6^A abundance in cellular mRNAs enriched from total RNA upon depletion of METTL3 or METTL14 (Fig 1E). Collectively, we validated the knockdown of METTL3 and METTL14 in HeLa cells using independent orthogonal methods.

**Fig 2:**
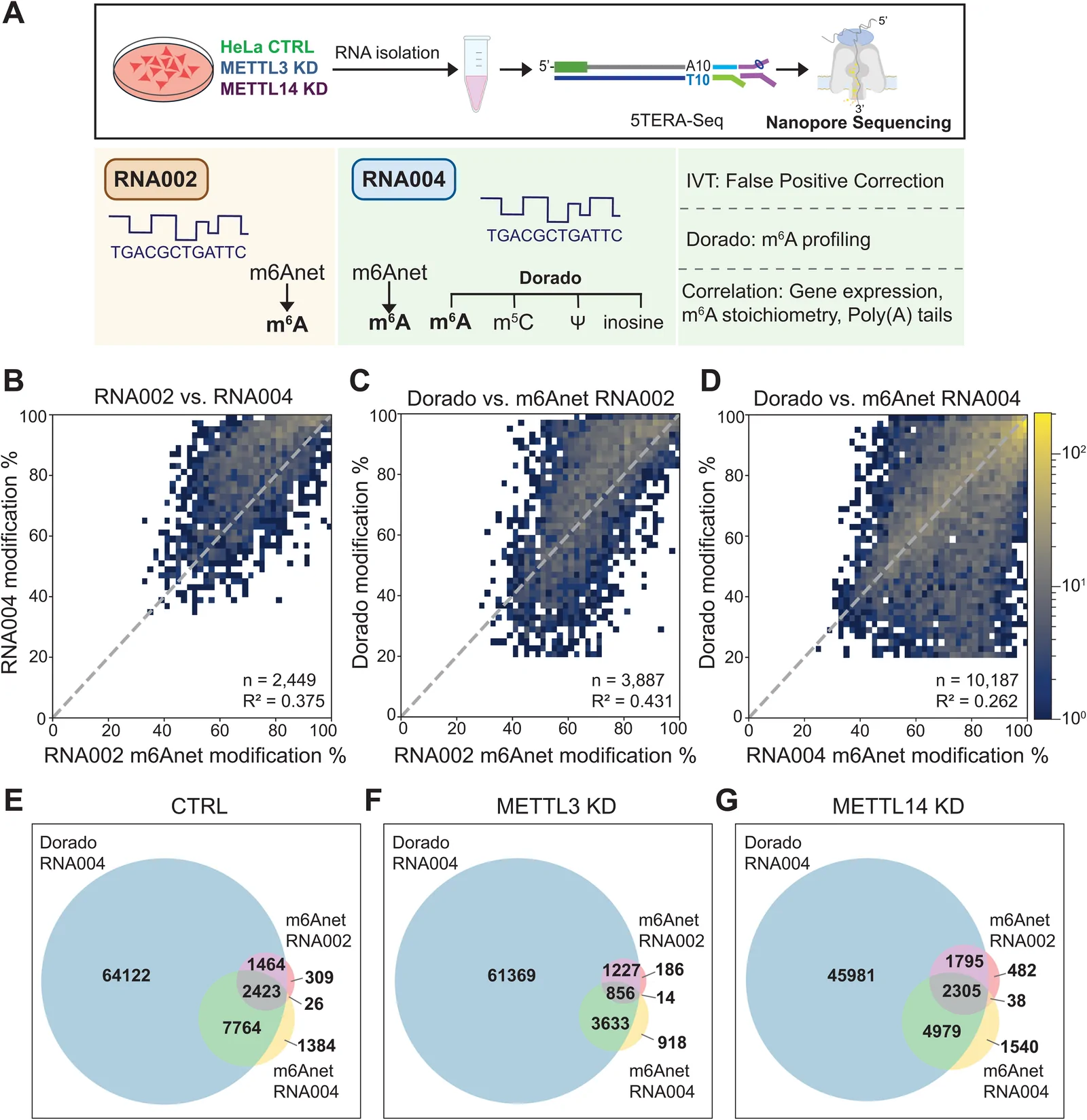
Comparison of valid m^6^A modification sites between RNA002 and RNA004 m6Anet with RNA004 ONT Dorado. (**A**) Overview of library preparation for direct Nanopore sequencing and modification detection models used for downstream analyses. (**B-D**) Scatter plots showing the correlation of m^6^A modification percentages in control (CTRL) between (**B**) RNA002 and RNA004 m6Anet, (**C**) RNA002 m6Anet and Dorado, and (**D**) RNA004 m6Anet and Dorado. The number of genomic positions with m^6^A modification calls (n) across all conditions and correlation values (R^2^) are depicted. (**E-G**) Venn diagrams depicting the number of genomic positions with valid m^6^A modification calls across control (CTRL, **E**), METTL3 knockdown (KD, **F**), and METTL14 KD (**G**) when called with RNA002 m6Anet, RNA004 m6Anet, or RNA004 Dorado.

To assess the performance of Nanopore dRNA-Seq in m^6^A detection, we performed dRNA-Seq using our previously established TERA-Seq protocol for ONT [33,34] on RNA extracted from HeLa CTRL, METTL3 KD, and METTL14 KD cells (Figs 2A and S1A). To ensure the generation of high-quality datasets, we assessed the integrity of total RNAs isolated from the various treatments with capillary electrophoresis prior to processing samples for library generation (S1B Fig). We first generated two biological replicates of each treatment using Nanopore RNA002 chemistry. Sequencing of the 6 libraries resulted in a total of ~6.6 million raw reads from all biological replicates with an average aligned N50 read length of ~1,100 bases (S2 Table). As expected, we detected high correlation between all biological replicates (Pearson correlation coefficient of 0.97-0.99; S1C Fig). Each biological replicate underwent independent processing to assess consistency among replicates before merging replicates for deeper downstream analyses. Next, we generated two biological replicates from each of the CTRL and METTL3/METTL14 KD cell lines using RNA004 chemistry under equivalent biological conditions. Sequencing of the libraries yielded substantial depth across all conditions with a total of ~101 million raw reads from all biological replicates, with an average aligned N50 read length of ~900 bases. Consistent with RNA002 datasets, we observed high correlations within the RNA004 datasets (Pearson correlation coefficient of 0.74-0.99) and across RNA002 datasets (0.81-0.94) (S1C Fig). To evaluate the levels of METTL3 and METTL14 transcriptome-wide, we extracted sequenced reads that map to METTL3 and METTL14 transcripts from RNA002 and RNA004 datasets and detected a reduction in CPM (count per million) values across datasets for both METTL3 and METTL14 compared to CTRL (S3 Table). These findings corroborate with our biochemical observations of METTL3/14 depletion in HeLa cells.

### Genome-wide detection of m^6^A modification using m6Anet and Dorado models with false positive-correction of putative m^6^A modified sites

To capture m^6^A methylation genome-wide, we applied neural network-based m^6^A calling models; m6Anet [26] for both RNA002 and RNA004 chemistries, and ONT Dorado for RNA004 datasets. m6Anet predicts the probability of the m^6^A modification for each candidate adenosine in each read and classifies a site as modified when the predicted probability exceeds a defined threshold, which is set to 0.033 by default for the human model. Conversely, Dorado depends on Modkit to identify the optimal threshold by sampling the distribution of probability predictions. Dorado assesses all adenosines in a read for m^6^A modification, and the reads are aligned to a reference genome using the splice-aware alignment mode of minimap2 [38]. Then, the “pileup” module in Modkit computes the modification ratio for all DRACH sites in the reference genome as the number of m^6^A predictions mapped to the reference DRACH site divided by the total number of all mapped adenosines.

To ensure specificity and robust comparative analysis between datasets, we further filtered the sites for m6Anet with a stringent 0.9 cutoff modification detection probability as previously recommended [26,31]. In contrast, for Dorado we constrained the parameters to include positions/sites with a coverage of at least 20 reads with reference-matching nucleotides aligned at the position and at least 20% predicted m^6^A-modification occupancy and reported as “valid m^6^A sites”. These criteria were selected based on our rigorous false-positive rate observed using a set of synthetic oligonucleotides [29] and are dependent on both sequencing coverage and selected modification percentages. Assuming the highest false-positive rate of 0.0121 as the success parameter for a binomial distribution, our filtering criteria gives a probability of a false-positive putative modification site as 0.0000854758 or < 1 in 10,000 sites. These approaches substantially reduced the number of m^6^A sites analyzed while enhancing robustness. The accuracy of modification calling remains a critical consideration in dRNA-Seq analysis, as algorithms can exhibit sequence-specific biases. Our previous work established that IVT RNA is an essential negative control for understanding Dorado’s false modification calling tendencies [29]. This IVT RNA consists of only unmodified nucleotides. We analyzed all 262,144 (4^9^) possible 9-base sequence contexts within the genome. For each context, we calculated how often the algorithm incorrectly identified modifications in our unmodified control RNA. This created a comprehensive “reference table” of false-positive rates for every possible sequence context [29]. When applied to the RNA004 datasets, the false-positive adjustment substantially refined modification detection. Using Dorado and false-positive correction, we removed 3,890 spurious m^6^A calls (4.88% of 79,663 calls) in the CTRL dataset. We corrected 3,438 false-positive calls (4.87% of 70,523 calls) in METTL3 KD, and 3,144 (5.40% of 58,204 calls) in METTL14 KD datasets (S4-S6 Tables).

To evaluate the m^6^A modification calling performance of m6Anet and Dorado, we first assessed the correlation between m^6^A modification ratios reported by m6Anet from RNA002 and RNA004 and by Dorado from RNA004 in the CTRL datasets. We observed modest agreement between all calling models across CTRL datasets (R^2^ 0.262-0.431; Fig 2B-2D). We then extracted the shared and unique sites in CTRL from the RNA002 and RNA004 datasets and found that the total number of putative modified m^6^A sites increased substantially from 4,222 (m6Anet; RNA002) to 11,597 (m6Anet; RNA004). In contrast, after false-positive correction, Dorado and RNA004 reported 75,773 m^6^A positions (Fig 2E). Applying the Jaccard index across the two models and datasets, we revealed a limited overlap between Dorado and RNA002 datasets (0.05) and a modestly higher similarity between Dorado and RNA004 datasets, as well as between RNA002 and RNA004 datasets using m6Anet (0.12-0.16) (S7 Table). This suggests that m6Anet likely produces a more restrained set of modification calls, consistent with the algorithm’s dependence on pre-processing of reads through a method called resquiggling, while a pure signal-based approach such as Dorado generally produces a larger range of callable sites. Despite the differences in the total number of called m^6^A sites between the two models, the majority of m6Anet-identified putative m^6^A sites from the RNA002 (92.07%) and RNA004 (87.84%) datasets are also reported when using Dorado (Fig 2E and S7 Table).

To investigate the impact of METTL3/14 depletion on m^6^A deposition, we first evaluated the calling performance of m6Anet for the RNA002 and RNA004 datasets by applying the same filtering parameters to include ≥ 20 reads coverage and ≥ 20% modification ratio. When we examined unique m^6^A sites in METTL3/14 knockdowns captured in RNA002 and RNA004 datasets, we identified a greater number of total putative m^6^A sites scored by Dorado (67,085, METTL3 KD and 55,060, METTL14 KD) compared to m6Anet (2,283 for RNA002 and 5,421 for RNA004, METTL3 KD; and 4,620 for RNA002 and 8,862 for RNA004; METTL14 KD) (Fig 2F and 2G). Importantly, we provide the first evidence for an overall reduction of total putative m^6^A sites in m6Anet after depletion of METTL3/14 compared to CTRL, and a more pronounced reduction in the total number of m^6^A sites scored by Dorado in both methyltransferase knockdowns, with 67,085 in METTL3 KD and 55,060 in METTL14 KD compared to 75,773 putative m^6^A sites in CTRL (Figs 2E-2G, S2A and S2B). Using the false positive-corrected Dorado sites, we extracted unique and shared sites between the three datasets and identified 44,038 m^6^A sites across all three conditions (S2C Fig). The Jaccard index revealed substantial overlap across all datasets (CTRL and METTL3 KD (0.59), CTRL and METTL14 KD (0.58), METTL3 KD and METTL14 KD (0.62)) (S7 Table). To assess the impact of methyltransferase depletion on global m^6^A levels, we used the false positive-corrected m^6^A sites identified using Dorado. Filtering for valid m^6^A-modified DRACH sites in CTRL, we report a modest global reduction in m^6^A methylation levels in the merged biological replicates of METTL3 KD (18.1%) and METTL14 KD (24.3%) datasets. We also calculated the per-site modification changes and report ~10% reduction for METTL3 KD and ~12% for METTL14 KD. Notably, the global reduction of m^6^A levels is significantly less as compared to our m^6^A dot blot results (Fig 1E). However, dRNA-Seq m^6^A calling and the m^6^A dot blot assay evaluate fundamentally different aspects of m^6^A biology, thus their outputs are not directly comparable. While the m^6^A dot blot is a semi-quantitative bulk assay, reflecting the total abundance of m^6^A, ONT dRNA-Seq provides site-level estimates of m^6^A modification probability [39,40]. Collectively, our data provides the first evidence of reduction of the total number of m^6^A-modified sites upon METTL3/14 depletion.

To gain further biological insights and capture m^6^A across all sequence contexts, we focused on RNA004-generated datasets with the false positive-corrected Dorado m^6^A sites for all downstream analyses. Our sequenced libraries displayed variable depth between the three conditions (S2 Table). To eliminate any biases due to differences in library depth, we randomly downsampled our primary aligned BAM files at different intervals of read depth ranging from 5.0 to ~29.5 million and compared them after site-level inference with ONT Modkit and false-positive correction. At every level examined, m^6^A modification counts exhibited a linear trend in all datasets and similar Jaccard index values across each read depth (S8 Table). To visualize the overlap among datasets, we generated a Venn diagram based on sites identified at the 20 million-read downsampling depth of merged biological replicates. We observed a consistent overlap of unique and shared Dorado m^6^A sites across all datasets before and after downsampling (S2C and S2D Fig). These findings show that our analysis remains robust to differences in read coverage. To ensure a consistent framework, we conducted all downstream analyses using the Dorado false positive-corrected m^6^A sites of both “all- and downsampled-aligned” reads for RNA004 datasets.

### m^6^A modification distribution in DRACH motifs of control and METTL3/14-depleted cells

To evaluate the authenticity of m^6^A sites reported by Dorado, we performed a metagene analysis of unique m^6^A sites across gene features (5’ untranslated region, 5’ UTR; coding sequence, CDS; and 3’ UTR). We report enrichment in the distribution of m^6^A sites near the end of the CDS and in the 3’ UTR (S3A and S3B Fig). Using publicly available m^6^A sites detected by m^6^A miCLIP from HeLa cells [41], we found that the resulting distribution of the m^6^A methylation profile in CTRL, METTL3 KD, and METTL14 KD is consistent with the distribution of miCLIP-identified m^6^A sites (S3C Fig). We further analyzed our Dorado CTRL dataset at ≥ 20 reads and ≥ 20% modification ratio against a publicly published HeLa GLORI dataset [42], a quantitative m^6^A mapping method, and found about 44% overlap of Dorado- and GLORI-m^6^A detected sites (S3D Fig).

**Fig 3:**
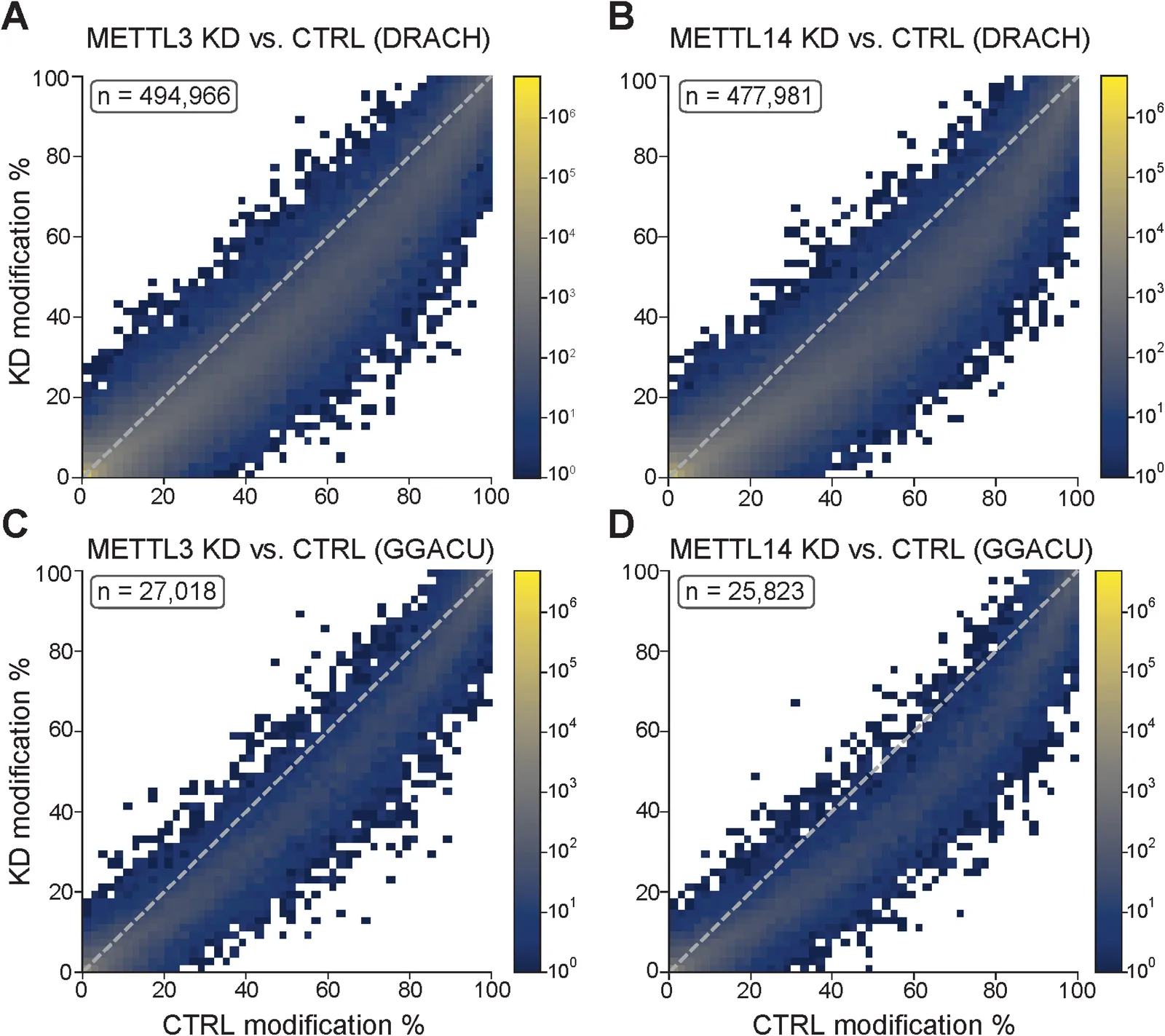
Comparison of m^6^A modification levels between control and METTL3/METTL14 depletion datasets and across DRACH motifs. (**A-D**) Scatter plots showing the correlation of m^6^A modification percentages between control (CTRL) vs. METTL3 knockdown (KD, **A**) and METTL14 KD (**B**) in DRACH motif, and CTRL vs. METTL3 KD (**C**) and METTL14 KD (**D**) in GGACU motif scored by Dorado. The number of genomic positions with valid m^6^A modification calls (n) scored by Dorado across all conditions are depicted. Each point represents a genomic position with valid read coverage in both conditions. Color intensity represents point density.

To further interrogate the dynamic changes of m^6^A levels, we categorized the modification sites into three sequence contexts based on established m^6^A consensus motifs: DRACH, GGACU, the most enriched m^6^A motif, as well as the non-DRACH sites. We expanded our analysis to include non-DRACH sites because, while DRACH is the primary motif for m^6^A deposition, prior literature suggests that this motif accounts for ~70% of all m^6^A sites [19,43]. Our initial comparative analysis of m^6^A levels using the Dorado m^6^A sites between CTRL and KD datasets revealed distinct patterns. When METTL3 or METTL14 is depleted, we observe a substantial reduction of m^6^A modifications in DRACH and GGACU motifs (Fig 3A-3D), without any impact on non-DRACH sites (S4A and S4B Fig), compared to CTRL. Applying similar parameters and categories to our downsampled datasets, we observed consistent trends between CTRL and METTL3/14 KD datasets (S4C-S4H Fig). Next, we analyzed the frequency of m^6^A modified sites for DRACH motif 5-mers in all three conditions. We detected a similar enrichment of motifs in all three datasets, with the highest enrichment in GGACU, as previously reported [3,19] followed by GGACA, GAACU, and AGACU (S3E-S3G Fig). Notably, we observed a reduction in the total number of m^6^A sites reported with the top 10 5-mers in METTL3 KD and METTL14 KD datasets compared to CTRL, consistent with the depletion of the writer enzymes. The reduction was more prominent for METTL14 KD dataset.

**Fig 4:**
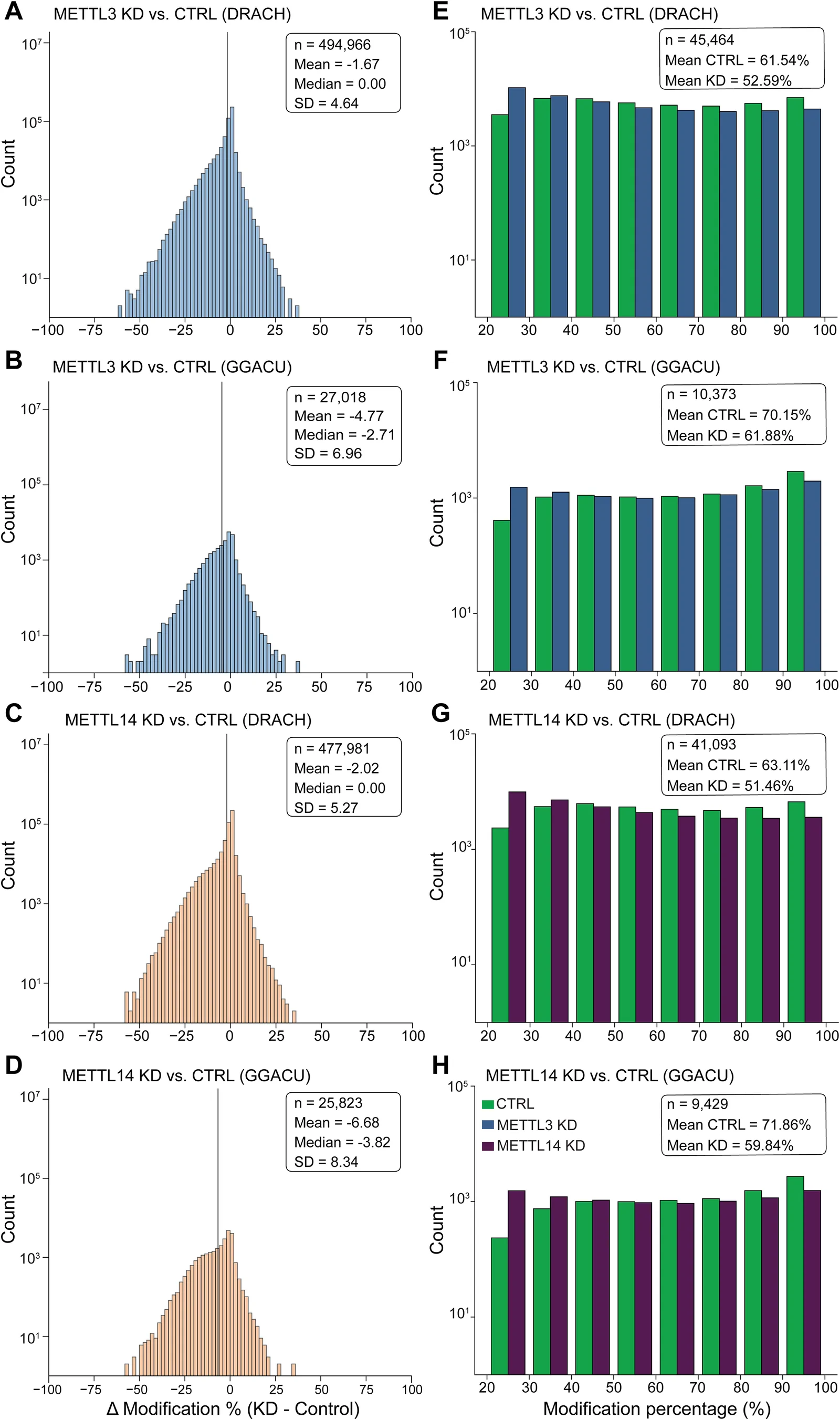
Distribution of the modification percentage of m^6^A between control and METTL3/14 knockdowns datasets across DRACH motifs. (**A-D**) Histograms showing the difference in m^6^A modification percentage (Δ Modification %) between knockdown (KD) and control (CTRL) conditions and combined site differences for (**A, C**) METTL3 KD and METTL14 KD vs. CTRL in DRACH motifs, and (**B, D**) METTL3 KD and METTL14 KD vs. CTRL in GGACU motifs. Negative values indicate decreased m^6^A levels upon knockdown. The number of sites (n), mean and median differences, and standard deviation (SD) are shown for each distribution. (**E-H**) Histograms showing the distribution of m^6^A modification percentage between knockdown (KD) and control (CTRL) conditions and combined site differences for METTL3 KD and METTL14 KD vs. CTRL in DRACH motifs (**E, G**), and METTL3 KD and METTL14 KD vs. CTRL in GGACU motifs (**F, H**). Only sites with valid coverage in both conditions are included.

To precisely measure the contribution of METTL3 and METTL14 to the genome-wide m^6^A modification in the context of DRACH and GGACU motifs, we calculated site-wise changes in modification levels between CTRL and METTL3/14 KD datasets. For each position, we subtracted the percentage of m^6^A modification in KD datasets from the modification percentage of the CTRL, generating “delta modification” values. While we obtained a slight overall reduction in the modification mean values for METTL3 KD (1.67%) and METTL14 KD (2.02%) in the DRACH motif, we observed a higher reduction in METTL3 KD (4.77%) and METTL14 KD (6.68%) in the GGACU motif context (Fig 4A-4D). Importantly, upon depletion of METTL3 or METTL14, we observed no changes in non-DRACH sites (S5A and S5B Fig), with an overall indistinguishable trend in the downsampled datasets (S5C-S5H Fig). Subsequently, we analyzed the percentages of modification distribution by motif type across genes and found a gradual reduction in the modification frequency in highly modified genes for both METTL3 KD and METTL14 KD datasets compared to CTRL for both the DRACH and GGACU motifs (Fig 4E–4H), while no noticeable changes were observed in non-DRACH motifs (S6A and S6B Fig). We observed similar distribution trends in downsampled datasets (S6C-S6H Fig).

**Fig 5:**
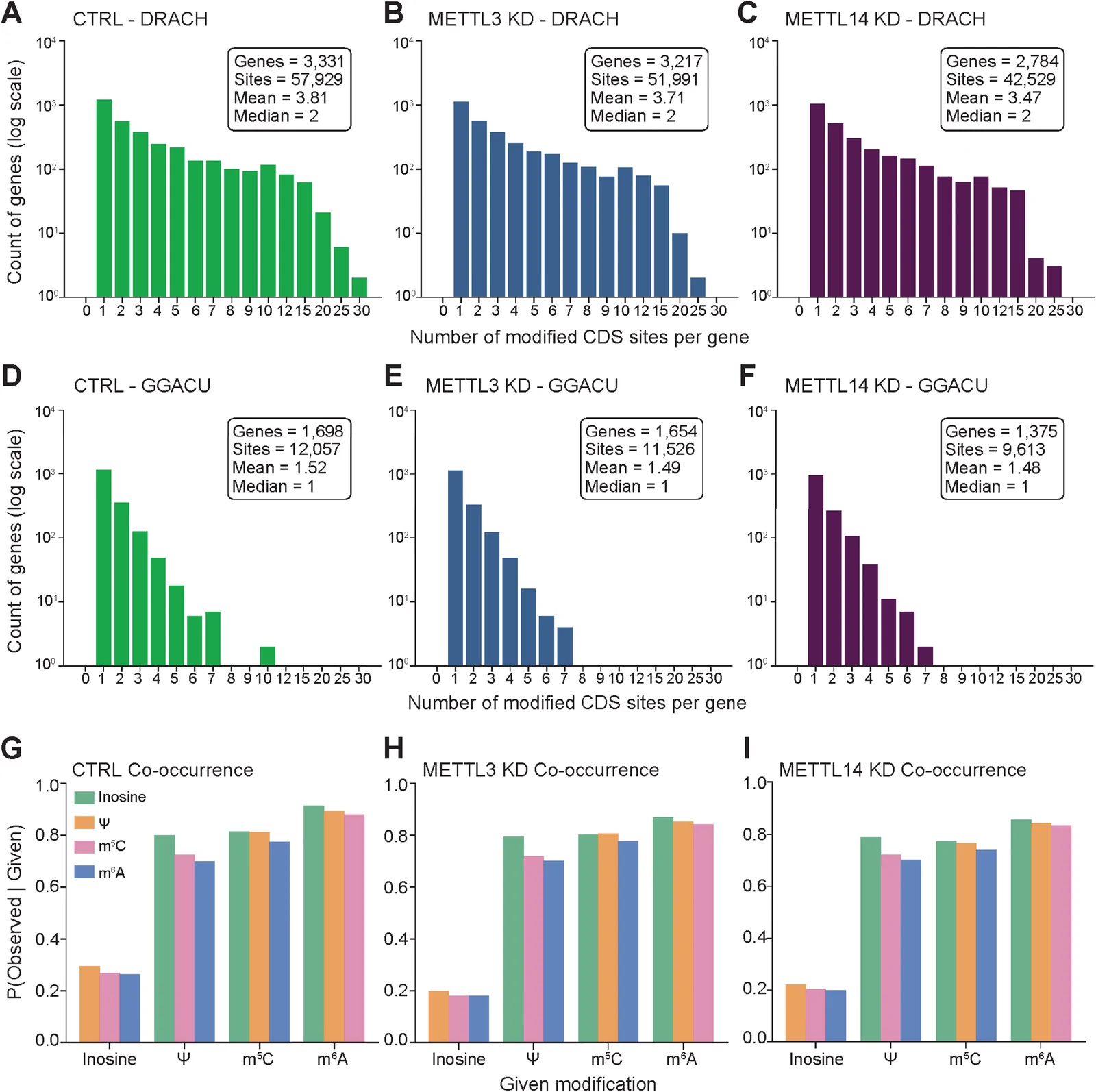
Distribution of unique modified coding sequence sites per gene and co-occurrence of m^6^A modifications across control and METTL3/14 knockdown datasets. (**A-F**) Histograms showing the frequency of genes containing different numbers of unique modified sites in coding sequence (CDS) across DRACH (**A-C**) and GGACU (**D-F**) motifs. Y-axes are log-scaled to visualize the full range of the distribution. (**G-I**) Bar plots showing the probability of observing a given modification type (inosine; pseudouridine, Ψ; 5-methylcytosine, m^5^C; and N^6^ methyladenosine, m^6^A) when another modification is present outside of a 5-mers window from that genomic position in control (CTRL) (**G**), METTL3 knockdown (KD) (**H**), and METTL14 KD (**I**) datasets. The x-axis indicates the given modification, while bars represent the probability of observing each modification type (Observed/Given) at co-occurring sites.

To assess the prevalence of m^6^A methylation across genes, we analyzed the distribution of uniquely modified sites per CDS relative to the total number of m^6^A sites per transcript in METTL3 KD and METTL14 KD versus CTRL, focusing on DRACH and GGACU motifs. While most genes have 1–5 modified sites, some genes have up to 15 modified m^6^A sites, and a small group of genes report up to 25 putative m^6^A sites at DRACH motifs in all datasets (Fig 5A-5C) and to a much lesser extent in non-DRACH motifs (S7A-S7C Fig). We detected a gradual reduction of highly modified sites in METTL3/14 KD compared to CTRL (Fig 5A-5C), and fewer m^6^A-modified sites per CDS in GGACU motifs (Fig 5D-5F), indicative of the specificity of m^6^A reduction following METTL3/14 KD. This distribution is mirrored in the downsampled datasets (S7D-S7L Fig). Collectively, these findings further demonstrate the robustness of m^6^A detection and the specificity of our METTL3/14 knockdowns.

### Co-occurrence of m^6^A with other RNA modifications

Dorado can detect multiple RNA modifications, including m^6^A, m^5^C, Ψ, and inosine. Therefore, we sought to interrogate if knockdown of METTL3 and METTL14 and reduction of m^6^A levels can influence the occupancy of m^5^C, Ψ, or inosine near m^6^A. We extracted all potential modifications called by Dorado (S4-S6 Tables) and calculated the co-observation probability between pairs of modifications using uniquely modified CDS sites per gene. Here, we defined the co-observation rate as the proportion of genes with a given modification X, and a given modification Y on the gene body. Then, we calculated the pairwise co-observation rate for the four modifications. We considered modifications outside of a 5 nucleotide (nt) window in both directions of a given modification, and all modification calls that occurred within 5 nt of each other were excluded from this analysis to limit the impact of modification-associated miscalls. The window size is based off the assumption that the approximate pore size of the ONT RNA004 pore is 9 nt. By implementing the 5 nt exclusion window on either side (an equivalent kmer size of 11), we ensure that no two modifications occupying the same kmer-window disrupt the ionic current flow enough to confound the modification calls and thereby centering the putative modification site in a 9 nt context window. Using Dorado (v.1.4.0) we observed that the lowest global abundance of detected modified sites after we filtered for ≥ 20 reads coverage and ≥ 20% modification ratio with IVT-based false positive adjustment was for inosine followed by pseudouridine (S4-S6 Tables). While pseudouridine’s detected sites are overall low, its co-occurrence is comparable to that of m^6^A and m^5^C across all datasets (Fig 5G-5I). Because the analysis is performed at the gene level, the site count and gene-level co-occurrence are not tightly coupled. Of all four modifications, we found that inosine modifications had a modest lower co-observation probability with m^6^A in METTL3/14 KD datasets compared to the CTRL (Fig 5G-5I). Conversely, no clear difference of co-occupancy was detected with the other modifications. These observations demonstrate the power of ONT and the abilities of the Dorado model to faithfully detect multiple modifications, while highlighting the need for further validations and investigation into the biological significance of co-regulation of RNA modifications and mRNA abundance.

### Characterization of m^6^A methylation and its correlation with mRNA abundance and poly(A) tail length

One of the major functions of m^6^A is to promote mRNA decay [13,44]. To evaluate the impact of METTL3/14 depletion on mRNA levels, we quantified genes displaying ≥ 10% change in modification between CTRL and METTL3/14 KD and categorized these genes into three modification deciles (20%, 50%, and 90%), retaining only valid m^6^A sites (≥ 20 reads and ≥ 20% modification ratio) across the datasets. Next, we quantified the differences in modification ratios across the datasets, generated “mean delta” values, and selected a subset of genes whose “mean delta” values reflected lower m^6^A modification ratios in METTL3 KD or METTL14 KD compared to CTRL or higher modification ratios after METTL3 depletion. Based on these criteria, we selected a total of 15 genes and examined their RNA expression levels using quantitative PCR (qPCR) analysis (S9 Table). Intriguingly, we observed that depletion of METTL3 and METTL14 caused distinct changes in RNA expression levels. First, we noticed divergent responses with minimal up or downregulation of most targets in all decile categories following METTL3 depletion (S8A and S8B Fig). Conversely, we observed that METTL14 depletion induced a robust upregulation for several selected targets (S8A and S8B Fig). We also detected opposing trends in RNA expression levels for some targets in METTL3/METTL14 KD datasets.

When we categorized all genes by functional groups, we observed that the loss of METTL14 strongly affected genes encoding RNAs involved in transcription and translation regulation, including *SOX13*, *SF1*, and *EIF3A* consistent with m^6^A regulation of RNA stability [45,46]. Genes encoding RNAs involved in the cytoskeleton and trafficking, including *SHCBP1* and *PLEKHM2*, were altered at different levels, suggesting that the levels of some regulatory genes are also influenced by m^6^A methylation. In agreement with previously reported observations [47,48], we find that genes encoding modified RNAs with roles in metabolic and proliferative processes such as *MOGS*, *PFKM*, and *TBC1D8* were moderately stabilized. For each of these genes, we retrieved the canonical transcripts and mapped m^6^A sites onto their genomic coordinates. We demonstrated an overall reduction of m^6^A-modified sites in METTL3/14 KD compared to CTRL, consistent with their depletion (S9A-S9L Fig). Together, we detected gene-specific differential regulations by METTL3 and METTL14, indicating cooperation as well as division of labor within the m^6^A writer complex.

**Fig 6:**
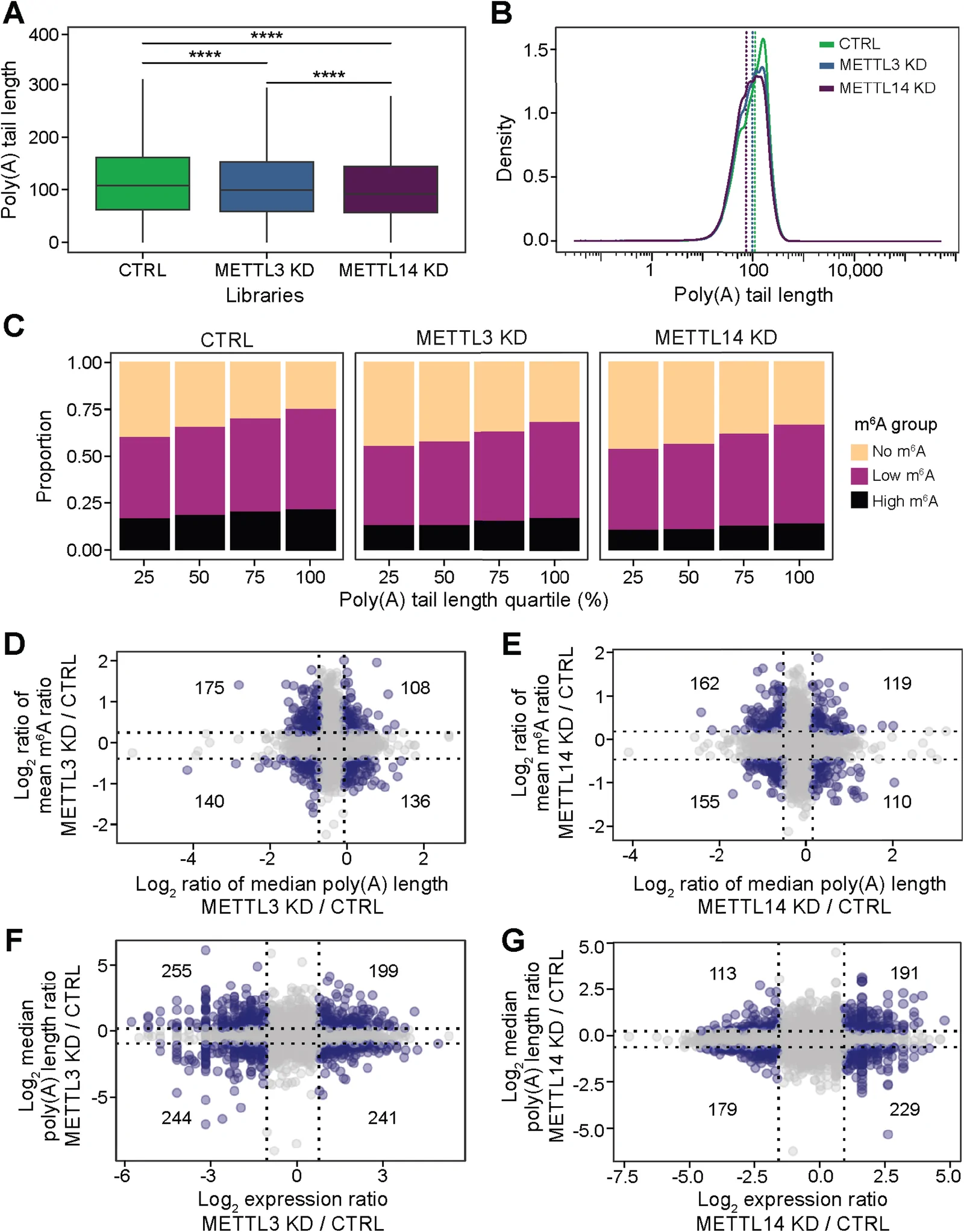
Correlations of m^6^A modification with poly(A) tail length and gene expression. (**A**) Box plot of median poly(A) tail lengths in control (CTRL) and METTL3/14 knockdown (KD) datasets (****p < 0.0001). (**B**) Density plot of poly(A) tail lengths from CTRL, METTL3 knockdown (KD), and METTL14 KD. (**C**) Stacked bar charts representing the percentage of poly(A) tail reads from each sample that falls into each poly(A) length quartile, with quartiles calculated based on control (CTRL), color-coded by mean m^6^A modification ratio based on Dorado calls across all sites on that gene. (**D, E**) Genes by log ratio of both median poly(A) length and mean m^6^A modification ratio in METTL3 knockdown (KD) vs. CTRL (**D**) and METTL14 KD vs. CTRL (**E**). (**F, G**) Genes by log ratio of both median poly(A) length and expression level (counts per million) in METTL3 KD vs. CTRL (**F**) and METTL14 KD vs. CTRL (**G**). Dotted lines indicate the mean log fold change ±1 standard deviation. m^6^A, N^6^-methyladensoine.

Growing evidence indicates that m^6^A modification and poly(A) tail length are directly correlated, with m^6^A-coupled RNA decay mediated by poly(A) tail shortening through recruitment of the CCR4-NOT complex [13]. Nanopore dRNA-Seq enables assessment of mRNA poly(A) tail lengths at single-molecule resolution. To comprehensively assess how METTL3/14 depletion influences mRNA abundance, we conducted three correlation analyses including poly(A) tails distribution, m^6^A stoichiometry, and gene expression across all datasets. We first estimated the poly(A) tail lengths in all datasets using Nanopolish [49]. We observed significant differences in tail lengths between the different datasets, with a median poly(A) tail of 112 nt in CTRL, 106 nt in METTL3 KD, and 96 nt in METTL14 KD (Fig 6A and 6B). We then divided the distribution of the poly(A) tail lengths in each dataset across quartiles defined by all poly(A)-containing reads in CTRL and binned transcripts by tail lengths (25% quartile, < 61 nt; 50%, 61 to < 108 nt; 75%, 108 to < 161 nt; and 100%, ≥ 162 nt). We observed a shift towards shorter poly(A) tails in both METTL3 KD and METTL14 KD compared to CTRL, with the first quartile containing more than 25% of poly(A) reads, and the last two quartiles containing less than 25% of poly(A) reads each in both KD datasets (S8C Fig). Overall, depletion of METTL3/14 led to a pronounced enrichment of transcripts harboring short-to-medium poly(A) tails.

To gain further insights into the distribution of poly(A) tail lengths relative to m^6^A modification ratios, we categorized m^6^A modification ratios based on stoichiometry: no m^6^A (no sites detected), low m^6^A (≤ 0.5), and high m^6^A (≥ 0.5). Next, we assigned m^6^A groups for each transcript based on the mean modification ratio across all sites within each transcript. For each poly(A) tail-containing read in each poly(A) quartile, we assigned the m^6^A category of the transcript to which the read maps. Comparison across all datasets for m^6^A groups and poly(A) tail quartiles revealed that the percentage of poly(A) tail reads from transcripts with no m^6^A remains mostly consistent across all datasets and poly(A) length quartiles. In METTL3/14 KD compared to CTRL, we observed that across reads with poly(A) tails in all tail length quartiles, there is a minor decrease in the proportion of reads from transcripts with high m^6^A modification, along with a corresponding increase in the proportion of reads from transcripts with no m^6^A (Fig 6C). This reflects a decrease in m^6^A modification in METTL3 KD and METTL14 KD, mainly from highly modified sites, which does not seem to disproportionately affect transcripts with poly(A) tails of any length. In all three conditions, there is a slightly higher proportion of reads from transcripts with high m^6^A stoichiometry in the longer poly(A) tail quartiles compared to the shorter poly(A) tail quartiles. This indicates that reads from transcripts with more m^6^A sites tend to have longer poly(A) tails, consistent with previous studies [50]. Next, we used the publicly available GLORI dataset from HeLa cells [42] and employed the same poly(A) quartile analysis with m^6^A stoichiometry. Consistent with our analysis using Dorado-called m^6^A, we found that m^6^A stoichiometry is lower across poly(A) tail length quartiles in METTL3/14 KD compared to CTRL (S8D Fig).

To evaluate the impact of altered m^6^A levels on poly(A) tail lengths, we analyzed all targets assessed by qPCR (S8A and S8B Fig) using Nanopolish to calculate the poly(A) tail lengths per read for each of the genes and binned reads by tail lengths using the same quartiles: 1–60 nt, 61–107 nt, 108–161 nt, and ≥ 162 nt. Among reads from most targeted transcripts in the 20% and 50% modification groups (except PLEKHM2), we see a shift toward shorter poly(A) tails in METTL3/14 KD (S10A and S10B Fig). Interestingly, among transcripts in the 90% modification and increased modification in METTL3 KD categories, we see more stable poly(A) lengths in METTL3/14 KD compared to CTRL (S10C and S10D Fig). These findings reveal the heterogeneity of poly(A) tails and support changes in poly(A) tail length in response to METTL3/14 depletion that may be influenced by m^6^A stoichiometry, suggesting a link between depletion of METTL3/14 and mRNA abundance. To assess the relationship between the poly(A) tail length, gene expression, and m^6^A levels after depletion of METTL3/14, we first calculated the mean m^6^A methylation ratio across all conditions using Dorado false positive-corrected m^6^A sites, the median poly(A) tail length, and transcript abundance (by CPM) for each gene. Next, we categorized the genes into four groups: increased poly(A) tail and m^6^A methylation in KD, decreased poly(A) tail and increased m^6^A methylation in KD, increased poly(A) tail and decreased m^6^A methylation in KD, and decreased poly(A) tail and m^6^A methylation. In METTL3 KD compared to CTRL, we detected 108 genes with increased poly(A) tail length and m^6^A methylation, 175 genes with decreased poly(A) tail and increased m^6^A methylation, 136 genes with increased poly(A) tail and decreased m^6^A methylation, and 140 genes with decreased poly(A) tail and m^6^A methylation (Fig 6D). In contrast, in METTL14 KD relative to CTRL, we detected 119 genes with increased poly(A) tail length and m^6^A methylation, 162 genes with decreased poly(A) tail and increased m^6^A methylation, 110 genes with increased poly(A) tail and decreased m^6^A methylation, and 155 genes with decreased poly(A) tail and m^6^A methylation (Fig 6E). To compare METTL3 KD and METTL14 KD, we quantified the overlap between each of the above gene sets. We detected 23 genes with increased poly(A) tail length and m^6^A methylation in both KDs, 37 genes with decreased poly(A) tail and increased m^6^A methylation, 32 genes with increased poly(A) tail and decreased m^6^A methylation, and 24 genes with decreased poly(A) tail and m^6^A methylation (S10 Table). These small numbers of overlapping genes correlate to Jaccard indexes under 0.15, indicating low similarities between gene sets. To examine the roles of transcripts whose poly(A) tails shorten with decreased m^6^A modification, we conducted a gene set enrichment analysis using Gene Ontology (GO) terms. While the top 10 significantly enriched terms by p-value in METTL3 KD included DNA metabolic processes, the 8 significantly overrepresented GO terms in METTL14 KD included mitochondrial and organelle-related terms (S8E and S8F Fig). Our findings suggest distinct yet overlapping effects of METTL3 and METTL14 depletion.

Next, we investigated the association between gene expression and poly(A) tail length to METTL3/14 KD. We extracted genes that are at least one standard deviation from the mean log fold change for both poly(A) tail length and gene expression. We categorized these genes into four groups: upregulated expression and increased poly(A) tail, downregulated expression and increased poly(A) tail, upregulated expression and decreased poly(A) tail, and downregulated expression and decreased poly(A) tail. Comparing METTL3 KD to CTRL, we identified 199 genes with upregulated expression and increased poly(A) tail length, 255 genes with downregulated expression and increased poly(A) tail length, 241 genes with upregulated expression and decreased poly(A) tail length, and 244 genes with downregulated expression and decreased poly(A) tail length (Fig 6F). In contrast, comparing METTL14 KD to CTRL, we identified 191 genes with upregulated expression and increased poly(A) tail length, 113 genes with downregulated expression and increased poly(A) tail length, 229 genes with upregulated expression and decreased poly(A) tail length, and 179 genes with downregulated expression and decreased poly(A) tail length (Fig 6G). In GO enrichment analysis of genes with upregulated gene expression and decreased poly(A) tail length, the 8 significantly overrepresented terms in METTL3 KD are related to DNA damage, DNA binding, and transcription, while the top 10 terms in METTL14 KD are largely related to ion binding and organelles (S8G and S8H Fig). Collectively, these findings indicate that METTL3/14-driven methylation changes can modulate the expression and poly(A) tail lengths of genes across diverse cellular pathways.

## Discussion

To our knowledge, this study provides the first joint analysis of both METTL3 and METTL14-mediated m^6^A modification using long-read sequencing. By integrating depletion models with an IVT control and m6Anet/Dorado models, we detected bona fide m^6^A modification sites. This study demonstrates the robustness of Nanopore dRNA-Seq for characterizing m^6^A modification at both the genome and transcriptome levels and at single-nucleotide resolution. Our findings reinforce recent studies and uncover new aspects of nuanced METTL3/14 regulatory functions in gene expression in human cells. We present multiple, independent lines of evidence that support the acute depletion of METTL3 and METTL14. Our high-quality dRNA-Seq data are consistent with other m^6^A profiling methods including miCLIP [41] and GLORI [42], providing further support for the reliability of dRNA-Seq for detecting m^6^A modification. Employing RNA002 and RNA004 chemistries, we generated a total of 12 biological libraries of poly(A)-enriched RNAs of CTRL, METTL3- or METTL14-depleted HeLa cells and evaluated the m^6^A calling performance of the m6Anet and Dorado models. We demonstrate the capabilities of both models in calling m^6^A with some discrepancies suggesting that methods originally developed for m^6^A detection for RNA002 may not translate directly to RNA004. Notably, Dorado detected more m^6^A sites compared to that of m6Anet in agreement with recent studies [28,31,51]. The adjustment of Dorado-reported m^6^A sites using an unmodified IVT control drastically reduced the total callable m^6^A sites; however, it provided robustness under all conditions. We observed consistency for m^6^A detection before and after downsampling of all RNA004 datasets, thus ensuring that any subtle changes detected after METTL3/14 depletion are due to biological differences rather than sequencing depth biases.

This study is subject to several technical limitations associated with current Nanopore dRNA-Seq approaches. Because library preparation relies on poly(A) selection, our dataset primarily captures polyadenylated transcripts and therefore underrepresents non-polyadenylated RNA species, including replication-dependent histone mRNAs, certain non-coding RNAs, circular RNAs, and polyuridylated transcripts [52–54]. Consequently, the observed relationships between m^6^A modification and poly(A) tail length should be interpreted within the context of poly(A)-containing RNAs rather than the full transcriptome. In addition, current ONT-based models for poly(A) tail analysis are optimized for adenylated tails and may incompletely resolve mixed or terminal uridylation events, which are increasingly recognized as important regulators of RNA stability and decay [55]. Despite these limitations, poly(A) selection was used to maximize coding transcript coverage and improve detection sensitivity for m^6^A-associated effects on mRNA metabolism. Future advances in Nanopore sequencing chemistry, basecalling algorithms, and rRNA depletion-based workflows with greater sequencing depth may provide a more comprehensive view of epitranscriptomic regulation across both polyadenylated and non-polyadenylated RNA populations.

Recent systematic evaluations of computational modification calling models delineate key strengths and limitations of existing algorithms, underscoring the urgent need for standardized benchmarking to ensure robust results, while also providing practical guidance for threshold selections [28,31,32]. Moreover, previous studies have shown that dRNA-Seq with the RNA002 chemistry is suitable for epitranscriptomic profiling using METTL3 depletion [32], while the integration of the RNA004 chemistry with dRNA-Seq has a clinical utility by validating the loss of RNA methylation in a patient carrying truncating mutations in the methyltransferase METTL5 [31]. However, most prior assessments focused on comparison to published datasets, use of synthetic controls, or relevant biological sources independently, rather than jointly. By integrating knockdowns of both METTL3 and METTL14 with two chemistries and their compatible calling models, stringent filtering, IVT control, and rigorous validations, our study mitigates prior limitations and reveals new biological insights. In this study, we find that METTL3/14 depletion resulted in a slight reduction in global m^6^A levels (~18–24%). This is constrained by sequencing depth, coverage, and model sensitivity. Consequently, partial reductions in methylation following METTL3 depletion may appear attenuated in ONT analyses, particularly for low-stoichiometry or low-coverage sites, despite substantial global decreases detectable by biochemical and quantitative assays such as dot blot or liquid-chromatography-tandem mass spectrometry [40]. Nonetheless, we observed pronounced global decreases in the total number of m^6^A sites in coding sequences, and across genes categorized based on m^6^A stoichiometry, with the highest reduction depicted in groups of genes with highly modified sites in agreement with a recent study [50]. We find that the DRACH GGACU motif displayed the greatest reduction, consistent with its well-documented enrichment of m^6^A, supporting the specificity of m^6^A-mediated METTL3/14 deposition.

m^6^A regulates many aspects of mRNA metabolism and adds considerable sophistication to gene regulation provoking different mRNA fates. Emerging studies reveal that this modification can influence poly(A) tail dynamics, occasionally promoting accelerated deadenylation and subsequent decay, or alternatively stabilization of transcripts by inhibiting deadenylation. Binding of YTHDF2 to m^6^A-containing RNAs and recruitment of the CCR4–NOT deadenylase complex accelerates poly(A) shortening initiating mRNA decay [13]. Conversely, increasing evidence indicates that m^6^A modifications stabilize transcripts and promote their translation by preventing deadenylation via direct binding of m^6^A readers to modified transcripts [17] or through the incorporation of m^6^A into poly(A) tails [56], thereby blocking the recruitment of the deadenylase complex. Moreover, studies have shown that m^6^A-containing mRNAs exhibited markedly longer poly(A) tails than unmodified counterparts [30,32]. Our findings tie to some of these observations, revealing a global reduction of poly(A) tails following METTL3 and METTL14 depletion. Transcripts with more m^6^A modifications exhibited longer tails, supporting the heterogeneity of poly(A) tail lengths in response to METTL3/14 depletion and suggesting that this heterogeneity may be influenced by m^6^A stoichiometry. At the transcriptome level, we find that m^6^A-modified transcripts harbor short-to-medium poly(A) tails after depletion of METTL3/14. Intriguingly, METTL3/14 depletion induces intricate regulation of modified transcripts, with downregulation of a subset in METTL3 KD (consistent with known m^6^A-mediated mRNA stabilization [57]), upregulation of other subsets, and predominant upregulation in METTL14 KD. This discrepancy may be attributed to variation in m^6^A modification ratios or the occupancy of sites in different genic elements (CDS and/or UTRs) in each KD dataset. Alternatively, it could arise from the de-repression of modified transcripts by METTL14 knockdown, possibly through reduced methylation-coupled decay or feedback activation. Another possible explanation for the division of labor between METTL3 and METTL14 is that, while METTL3 provides catalytic activity, METTL14 may contribute more strongly to RNA substrate recognition or recruitment of the writer complex to specific transcript or structural contexts [58]. Consequently, depletion of METTL14 may impair methylation targeting efficiency across subsets of RNAs more specifically than depletion of METTL3 alone. Together, these findings support an emerging model in which METTL3 and METTL14 exert distinct regulatory effects on m^6^A deposition. Although METTL3 and METTL14 are well described necessary partners, there are a number of studies supporting non-redundant roles of the two proteins. One of the most well-known examples is the role of METTL3 in promoting the translation of a variety of transcripts, particularly in cancer, independent of its role in m^6^A catalysis [35,36]. Distinct effects of METTL3 and METTL14 depletion have also been described in the context of stemness maintenance in mouse embryonic stem cells [6,7]. METTL14 led to decreased nascent RNA synthesis, while METTL3 depletion resulted in transcriptional upregulation. Concordant with other studies, our study also reveals that genes of altered m^6^A levels and poly(A) tails are enriched in common and distinct biological processes, suggesting functional specialization between METTL3 and METTL14. This intricate regulation of modified transcripts by METTL3/14 suggests new directions for further exploration.

Multiple modifications along an mRNA portray an additional layer of complexity. However, the impact of multiple RNA modifications in the regulation of mRNA fate is still lacking. Leveraging Dorado, we evaluated the other putative RNA modifications (m^5^C, Ψ, and inosine). While the depletion of the m^6^A-modifying METTL3/14 did not alter the occupancy of most modifications in the vicinity of m^6^A, we observed a slight reduction in the occupancy of inosine. It was shown that inosine (A to I editing), is more likely to occur on transcripts that do not contain m^6^A, and that depletion of m^6^A writers drastically alters the A to I landscape. m^6^A destabilizes double-stranded RNA, decreasing accessibility of ADAR enzymes [59,60]. Additionally, it was shown that m^6^A modulate the ADAR1 through the binding of YTHDF1 to a conserved m^6^A site on the ADAR1 transcript promoting its translation [61–63]. However, other studies revealed distinct outcomes using different cell lines demonstrating that downregulation of METTL3 does not affect ADAR1 levels unless the interferon response is stimulated [61]. It is possible that a knockdown of METTL3 (and loss of m^6^A) could lead to a loss of translation of ADAR1, leading to a decrease in inosine levels. Such nuanced m^6^A and inosine modification dynamics offer a compelling direction for further studies. Validating these modifications using orthogonal techniques such as chemical assays, and biological, or synthetic controls will increase confidence in the accuracy of modification detection using Nanopore. Collectively, our study reveals that altered m^6^A levels impacts the gene expression and poly(A) tail dynamics of modified transcripts, establishing a valuable resource for delineating the role of METTL3/14 in mRNA stability. At the transcriptome level, depletion of methyltransferase enzymes correlates with shorter poly(A) tails, yet the consequences of the depletion vary across transcripts. This variability highlights the multifaceted post-transcriptional roles of m^6^A. Our METTL3/14-based mapping expands the utility of Nanopore to investigate how RNA modifications influence mRNA fate.

## Methods and materials

### Cell lines

HeLa cells were obtained from ATCC (CCL-2.1). Cells were maintained in DMEM (Gibco) supplemented with 10% FBS (Sigma) and 1% Penicillin-Streptomycin (Gibco), at 37°C in 5% CO2. Cells were free of mycoplasma.

### siRNA-mediated knockdown

To achieve a knockdown of METTL3 and METTL14 in HeLa cells, cells were transfected with Human targeting SMARTpool small interfering RNAs (siRNAs) at a final concentration of 20 nM for METTL3, and 25 nM for METTL14 or non-targeting control siRNAs (negative control) (Dharmacon), using Lipofectamine RNAiMAX (ThermoFisher) following the manufacturer’s protocol. Transfection was repeated after 24 hours. Forty-eight hours post-transfection, cells were harvested and lysed for either RNA isolation or cytoplasmic extract collection.

### RNA extraction

Total RNA was extracted with immediate lysis in TRIzol Reagent (ThermoFisher) and treated with DNase (Promega), as previously described [64,65]. The integrity of total RNA was assessed on an Agilent Bioanalyzer prior to each library preparation (S1B Fig).

### Immunoblotting

Cell lysate was prepared in lysis buffer (50 mM Tris-HCL (pH 7.5), 150 mM NaCl, 1% NP-40, 0.1% SDS, 0.5% sodium deoxycholate, and complete EDTA-free protease inhibitors (Roche)). Total protein concentration was determined through Bradford assay (ThermoFisher) per manufacturer’s instructions. Immunoblotting was performed as previously described [66]. The membranes were imaged using Azure Biosystems 300Q Imager. Quantification of the blots was performed with ImageJ software. The following antibodies were used: anti-METTL3 (1:8,000; Bethyl, A301-567A-T), anti-METTL14 (1:15,000; Proteintech, 26158–1-AP), anti-GAPDH (1:15,000; Sigma-Aldrich, G8795), anti-β-actin (1:15,000; Cell Signaling, 3700T), anti-m^6^A (1:1,000; Cell Signaling, 556593S), anti-MYC (1:5,000; Cell Signaling, D84C12), anti-DICER1 (1:7,000; Bethyl, A301-936A-T), HRP-conjugated goat anti-rabbit (Azure Biosystems, AC2114), and HRP-conjugated goat anti-mouse (Azure Biosystems, AC2115).

### Library preparation with TERA-Seq and Nanopore sequencing

Biological libraries were prepared using Nanopore dRNA-Seq kits (SQK-RNA002 and SQK-RNA004, ONT) following manufacturer’s protocol with modifications, as described previously [33,34]. Briefly, poly(A) mRNA was enriched from total RNAs using Oligo-dT dynabeads (ThermoFisher) following manufacturer’s protocol. A 5′ adapter (5TERA) was ligated on the beads using T4 RNA ligase 1 (NEB) plus 12.5% polyethylene glycol. ONT adapters (provided in the ONT kit) were ligated using T4 DNA Ligase (NEB) and the Quick Ligation Reaction Buffer (NEB), as previously described [33,34]. The first strand of cDNA was synthesized using SuperScript III reverse transcriptase (ThermoFisher; for RNA002) and Induro reverse transcriptase (NEB; for RNA004) per the ONT protocol’s instructions. Cleanup and capture of the RNA-cDNA was performed using RNAClean XP beads (Beckman Coulter). Sequencing was performed on the MinION or PromethION 2 Integrated devices using R9.4 flow cells (FLO-MIN106, RNA002, two biological replicates; and FLO-MIN004RA, RNA004 (biological replicate 1); and FLO-PRO004RA (biological replicate 2)) and the standard MinKNOW settings recommended by ONT for a 72-hours run.

### SELECT detection method

The SELECT method was performed following Xiao et al. 2018 [37] with minor modifications. Briefly, a total of 2.2 µg total RNA was mixed with 40 nM up primer, 40 nM down primer, and 5 mM dNTPs in 1X rCutSmart buffer (NEB) in a total volume of 17 µl. The RNA and primers were annealed through a temperature gradient, and the reactions were combined with a 3 µl mixture containing 0.01 U Bst 2.0 DNA polymerase (NEB), 0.5 U SplintR Ligase (NEB), and 10 nmol ATP (NEB). qPCR was performed using PowerUp SYBR master mix (ThermoFisher), 200 nM qPCR forward primer, and 200 nM qPCR reverse primer. Incubations were performed as described previously [37]. Fluorescence was collected at a ramping rate of 0.05°C/s. For quantification, the comparative delta Ct (ΔCt) method was applied, in which Ct values of m^6^A modified or unmodified sites were subtracted from the Ct value of each corresponding transcript then converted to 2^−ΔΔCt^ to obtain expression ratios. The statistical analysis was performed, and graphs were generated using Graphpad Prism (v10.5.0). The primers are listed in S1 Table.

### m^6^A dot blot

After transfection of control, METTL3, or METTL14 SMARTpool siRNAs in HeLa cells as described earlier, poly(A) mRNA was enriched using Oligo-dT beads following the manufacturer’s protocol. Following enrichment of polyadenylated mRNAs, 75 ng of each sample was spotted onto a Hybond-N+ membrane (GE Healthcare). RNA was crosslinked to the membrane via UV irradiation (1200 x 100 mJ/cm2). The experiment was performed in duplicate, with one membrane used for methylene blue staining and the other membrane for antibody probing. As a loading control, methylene blue staining was performed for 5 min using 0.02% methylene blue (Sigma-Aldrich) in 0.3 M sodium acetate. The other membrane was blocked directly as described previously [66]. This was followed by incubation with the anti-m^6^A antibody (Cell Signaling) and an HR-conjugated goat anti-rabbit antibody (Azure Biosystems). The membrane was exposed with Radiance ECL (Azure Biosystems) and imaged using an Azure Biosystems 300Q Imager.

### Quantitative real-time PCR (qRT-PCR)

Total RNA was extracted from HeLa cells with TRIzol, as previously described [65]. cDNA was synthesized from 250 ng of total RNA using Superscript III Reverse Transcriptase (ThermoFisher) following the manufacturer’s protocol. qPCR was performed using the PowerUp SYBR Green Master Mix (ThermoFisher) following the manufacturer’s protocol in a QuantStudio 3 System (Applied Biosystems). Each reaction was performed in triplicate. The primers are listed in S1 and S9 Tables. To evaluate transcript expression levels across treatment groups, the comparative ΔCt method was applied, in which Ct values of samples are subtracted from the Ct value of GAPDH and converted to 2^−ΔΔCt^ to obtain the expression ratios relative to GAPDH.

### Data analysis

#### Alignment and postprocessing.

Raw BAM files were converted to FASTQ format using samtools [67] and aligned to the GRCh38/hg38 reference genome using minimap2 (v2.26-r1175) [38] with splice-aware parameters optimized for RNA (-ax splice -uf -k14). BAM files were sorted using samtools (v1.19.2) [67] and filtered to retain only primary alignments (-F 2308). Modification tags (MM and ML) were preserved throughout processing. After alignment, sorting and filtering, the BAM files for both replicates were merged using samtools, prior to downstream processing with either m6Anet or Modkit.

### m^6^A calling with m6Anet and Dorado

To compare samples sequenced using the RNA002 chemistry, m6Anet (v2.1.0) [26] was used to detect m^6^A in dRNA-Seq data. Reads were aligned to the Gencode v47 transcript reference using minimap2 [38]. The aligned BAM file was subsequently sorted, filtered to include only primary reads, and indexed using samtools. Nanopolish [49] eventalign was used to produce event-level alignments for use with m6Anet (nanopolish eventalign --scale-events --signal-index). We used m6Anet to identify putative m^6^A sites in the RNA002 datasets. m6Anet’s HCT116_RNA002 model was used for inference. m^6^A sites were called in the RNA004 datasets using m6Anet as well. The same series of steps was followed to generate these calls, except that f5c [68] was used to produce the event-level alignments since Nanopolish has not been updated for RNA004 data.

nanopolish eventalign

f5c eventalign --rna --signal-index --scale-events

m6anet dataprep --eventalign

m6anet inference --pretrained_model {HCT116_RNA002, HEK293T_RNA004} --num_iterations 25

All modifications including m^6^A were called using Dorado (v1.4.0) (https://github.com/nanoporetech/dorado) with the 8_mods mode to identify m^6^A, m^5^C, Ψ, and inosine, and all 2’ O-methyl modifications simultaneously. Site-specific modification occupancy was calculated using the ONT Modkit (v0.6.1) pileup function. To ensure valid coverage, only positions with ≥ 20 reads were retained for downstream analysis. The “delta” or difference in occupancy, was calculated by subtracting the false positive-corrected modification percentage in CTRL from the same value for each respective METTL KD.

Dorado basecaller sup rna004_sup@v5.3.0_pseU_2OmeU@v1 rna004_sup@v5.3.0_inosine_m6A_2Om

eA@v1 rna004_sup@v5.3.0_m5C_2OmeC@v1 rna004_sup@v5.3.0_2OmeG@v1

modkit sample-probs –sampling-frac 0.10

modkit pileup –sampling-frac 0.10 –filter-threshold A: –filter-threshold C: --filter-threshold G: --filter-threshold T:

### Modification occupancy correction and re-filtering with whole genome IVT data

To adjust the data, a 9-mer false-positive rate “reference table” was used from Tzadikario et al. 2025 [29]. The reported modification occupancy was calculated of each site in the Modkit pileup. After subtracting the false-positive rate, the sites were filtered to ensure a minimum coverage of 20 valid modified reads. To measure the global modification rate, the pooled modification rate in the RNA004 merged biological replicates was calculated as the bulk-RNA measurement (∑n_mod/ ∑n_valid_cov). The per-site modification delta was calculated as the average change in false positive-corrected modification percentage.

### Downsampling and modification deciles analyses

After alignment to the GRCh38 reference genome and filtering for primary reads only, BAM files for each dataset were subsampled randomly at raw read counts ranging from 5,000,000–29,515,124 (S8 Table). At each subsampling depth, ONT Modkit and our false-positive correction were applied. After filtering for ≥ 20-read coverage and ≥ 20% false-positive-corrected modification ratio, overlap statistics were calculated. At the 20,000,000-read subsampling depth, sites were compared using a Venn diagram to visualize the overlap and was used for all downsampling analyses. To ensure valid modifications, all datasets were filtered to retain only positions where the CTRL false positive-corrected modification percentage was ≥ 20%. Sites were classified based on the following categories: DRACH motif (D[AGU]R[AG]H[ACU]), GGACU, or non-DRACH (all remaining kmers). False positive-corrected modification percentages were binned into 10% intervals (starting with 20–30% and ending with 90–100%). For each motif category and METTL knockdown comparison, grouped bar charts were generated to compare the distribution of modification percentages between the CTRL and KD conditions on a logarithmic scale. Mean modification percentages were calculated and displayed for each condition within each motif category.

### Co-occurrence calculations and DRACH motif analysis

Gene annotations were obtained from GENCODE v47 GTF files for gene-level analysis. To identify modifications in the CDS, positions were collapsed by gene ID, prioritizing annotations in the following order: UTR > CDS > start/stop codon > other exonic regions. Then, only CDS regions were considered. These annotations were used to count the total number of sites for different transcript types. Co-occurrence analysis examined modifications within genomic windows, excluding 5-mers around each site to prevent nanopore signal overlap. Data processing was performed using Python (v3.13) [69], with the pandas (v2.3.1), numpy (v2.1.2) [70], matplotlib (v3.10.5) [71] and seaborn (v0.13.2) [72] libraries. Motif classification (DRACH, (D[AGU]R[AG]H[ACU]); GGACU) was performed using regular expressions. These regular expressions were used to calculate the percentage distributions of each 5-mer among DRACH motifs.

### Gene annotation and CDS analysis

Gene annotations were obtained from GENCODE v47 GTF files. Each position was assigned a single feature type using a prioritization hierarchy (UTR > CDS > start/stop codon > other exonic regions), after which only CDS-annotated sites were retained for coding sequence analysis. These annotations were also used to quantify modification sites across different transcript types. For CDS distribution analysis, each condition (CTRL, METTL3 KD, and METTL14 KD) was processed independently. After annotation, datasets were filtered for false positive-corrected modification percentage ≥ 20%. Sites were classified into DRACH, GGACU, and non-DRACH motif categories. For each motif category and condition, the number of modified CDS sites per gene was quantified by grouping sites by GENCODE v47 gene identifier and counting occurrences within CDS features. CDS site counts per gene were binned using custom intervals (0–1, 1–2, 2–3, 3–4, 4–5, 5–6, 6–7, 7–8, 8–9, 9–10, 10–12, 12–15, 15–20, 20–25, 25–30). The distribution of genes by CDS site count was displayed on a logarithmic scale and plotted by condition and motif type.

### Transcript expression and correlations

Counts per million (CPM) for each gene in each sample was calculated using the cpm function from edgeR (v4.6.3) [73]. Pearson correlation coefficients were calculated for each pair of samples in R using CPM for each gene. CPM was extracted for METTL3 and METTL14 expression in each merged sample. For RNA004 samples, these values were normalized to CTRL before plotting.

### Metagene analysis and orthogonal datasets

Dorado- and miCLIP-called m^6^A sites were filtered for modification ratios of at least 0.1. Dorado m^6^A sites were also filtered to only include sites with ≥ 20 reads coverage. Genomic coordinates of sites were converted to transcriptomic coordinates of the longest transcript for each gene with the ensembldb (v2.32.0) [74] and EnsDb.Hsapiens.v86 (v2.99.0) R packages [75]. Metagene features (5’ UTR, CDS, and 3’ UTR) were mapped onto these positions using the Ensembl GRCh38 human genome annotation GTF file (v91) [74]. m^6^A sites that did not map to a metagene feature were removed from analysis. Lengths of each metagene feature were calculated, and distance from metagene feature start to m^6^A site was calculated. The relative metagene position was calculated as nucleotides from metagene feature start to m^6^A site, divided by length of metagene feature. Relative metagene positions were plotted for each sample using ggplot2 (v4.0.1) [76]. For comparison with the GLORI-Seq 1.0 from HeLa dataset [42], the chromosome, start position, end position, and methylation percentage were extracted under control conditions. The site-level comparisons were performed using our CTRL dataset using Dorado and positions with ≥ 20 reads coverage and ≥ 20% modification occupancy and false-positive correction. The overlap and unique sites were visualized using the matplotlib_venn (v1.1.2) venn2 package.

### Quantification of m^6^A and correlations with poly(A) tail lengths and gene expression

Poly(A) tail lengths for all poly(A)-containing reads in each RNA004 sample were plotted as a box plot. A t-test was used to determine significance between groups, with two biological replicates per group, with the R package rstatix (v0.7.2) [77] (Fig 6A). Density of poly(A) tail lengths was also plotted (Fig 6B). Poly(A) length quartiles were calculated from all poly(A)-containing reads in CTRL (1–60 nt, 61–107 nt, 108–161 nt, and ≥ 162 nt). Each poly(A) read in each of the three samples was assigned to the corresponding CTRL-based quartile. m^6^A values were assigned to each read based on the mean m^6^A modification ratio across all m^6^A-modified sites identified by ModKit. m^6^A groups across genes were defined as no m^6^A (no m^6^A sites identified), low m^6^A (mean modification ratio ≤ 0.5), and high m^6^A (mean modification ratio ≥ 0.5). The proportion of each sample in each combination of m^6^A bins and poly(A) quartiles was calculated and used to generate a stacked bar plot (Fig 6C). For each gene, the mean m^6^A ratio across all modified sites was calculated in each sample. The median poly(A) tail length was also calculated for each gene, and the CPM for each gene was calculated using edgeR (v4.6.3) [73]. Each gene was plotted by log ratio of both the calculated m^6^A value and the median poly(A) length in METTL3 KD and METTL14 vs. CTRL. Each gene was also plotted by log ratio of both CPM value and the median poly(A) length across datasets (Fig 6D-6G). Similarly, the processed data from published GLORI-Seq 1.0 in HeLa cells [42] was used to determine the mean m^6^A modification ratio across all modified sites for each gene. Genes were grouped by no m^6^A (no m^6^A sites identified), low m^6^A (≤ 0.5), and high m^6^A (≥ 0.5). The mean ratios and standard deviations across all genes of m^6^A modification ratios for METTL3 KD/CTRL and METTL14/CTRL were calculated. The mean and standard deviation of log fold change in both gene expression and the median poly(A) length by gene were also calculated for the same pairs of samples (S8D Fig).

### Gene Ontology analysis

Gene Ontology (GO) analysis was done on sets of genes over 1 standard deviation (SD) from the mean of both m^6^A ratio and poly(A) length ratio, and over 1 SD from the mean of both expression ratio and poly(A) length ratio. Gene overrepresentation analysis among GO was performed using the gost function from gprofiler2 (v0.2.3) on R (v4.5.0) [78].

### m^6^A sites mapping along genes

Libraries were downsampled to the same depth. For each selected gene, the canonical transcript based on the Gencode (v48) primary assembly was plotted using ggplot2 (v4.0.1). Positions with ≥ 20% m^6^A modification percentage and ≥ 20 reads coverage were plotted, with color indicating the modification ratio of the indicated position and each dataset (S9 Fig).

### Estimation of poly(A) length using Nanopolish

Poly(A) tail lengths were estimated for all RNA004 datasets using Nanopolish (v0.14.0). To convert Pod5 files to Fast5, pod5 convert to_fast5 was used. After basecalling, dRNA-Seq reads were indexed using “nanopolish index*”* to link the individual Fast5 files to their corresponding fastq files. The indexed reads were then processed using “nanopolish polya*”* to estimate poly(A) tail lengths based on the raw nanopore signal data. Reads labeled as *PASS* in qc_tag were retained for subsequent analyses. To estimate poly(A) tail length distribution for selected genes following METTL3/14 KD, a custom Python script with pandas (v2.2.2) was used. First, reads labelled *PASS* or *SUFFCLIP* and carrying a positive tail value were retained, and for each transcript the median tail length was calculated. Each valid read was assigned to one of three categories based on its poly(A) tail length: 1–60 nt, 61–107 nt, 108–161 nt, or ≥162 nt. For each gene and dataset, the number of transcripts in each of these three categories was normalized to the total number of transcripts for that gene/dataset pair to calculate the precise percentage of each category (S10 Fig). All plots were generated using the ggplot2 package in R (v4.0.1) [76].

## Supporting information

S1 Fig Library generation using TERA-Seq, RNA quality check, and libraries correlation. (A) Schematic of TERA-Seq for Oxford Nanopore Technologies (ONT). A10, poly(A) tail; T10, thymidine. (B) Bioanalyzer trace of a representative HeLa total RNA isolated from the control cells that was used for TERA-Seq library generation depicting ribosomal RNAs (28S and 18S). FU, Fluorescence unit. (C) Pearson correlation coefficients for RNA002 and RNA004 datasets.
(EPS)

S2 Fig Overlap of valid m^6^A modification sites between control and METTL3/14 knockdown. (A-D) Venn diagrams showing the number of genomic positions with valid m^6^A modification calls across control (CTRL), METTL3 knockdown (KD), and METTL14 KD samples scored by m6Anet for RNA002 with a probability of > 0.9 (A) and RNA004 (B) and using Dorado with ≥ 20 reads coverage and ≥ 20% modification after false-positive correction for the full (C) and downsampled (D) RNA004 datasets. The central region represents identified sites shared across all three conditions.
(EPS)

S3 Fig Feature distribution of unique m^6^A modifications across all transcripts and DRACH motifs. (A-B) Distribution of unique m^6^A modifications across feature types (5’ untranslated region, 5’ UTR; coding sequence, CDS; and 3’ untranslated region, 3’ UTR) was calculated for both the full datasets (all reads) (A), and the downsampled datasets (B). (C) Metagene plot illustrating the transcriptome-wide distribution of m^6^A across the feature types from publicly available miCLIP and RNA004 dRNA-Seq from control (CTRL), METTL3 knockdown (KD), and METTL14 KD. (D) Site comparison between publicly available GLORI HeLa control sample and CTRL HeLa false-positive corrected Dorado sites filtered for ≥ 20 sites and ≥ 20% modification percentage. DRS, direct RNA sequencing. (E-G) Counts of the top DRACH motif 5-mers in control (CTRL, E), METTL3 knockdown (KD, F), and METTL14 KD (G).
(EPS)

S4 Fig Comparison of m^6^A modification levels between the different conditions in non-DRACH and DRACH motifs. (A-H) Scatter plots showing the correlation of m^6^A modification percentages between METTL3 knockdown (KD) and METTL14 KD vs. control (CTRL) in full datasets (A, B) and downsampled datasets (C-H) in non-DRACH (C, D), in DRACH (E, F), and in GGACU motifs (G, H). The number of genomic positions (n) with valid m^6^A modification calls scored by Dorado across all conditions are depicted. Each point represents a genomic position with valid read coverage in both conditions. Color intensity represents point density.
(EPS)

S5 Fig Distribution of m^6^A modification level changes between METTL3/14 knockdown and control datasets across non-DRACH and DRACH motifs. (A-H) Histograms showing the difference in m^6^A modification percentage (Δ Modification %) between knockdown (KD) and control (CTRL) conditions for full datasets (A, B), and downsampled datasets (C-H) in non-DRACH (C, D), DRACH (E, F), and GGACU motifs (G, H). Negative values indicate decreased m^6^A levels upon knockdown. The number of sites (n), mean and median differences, and standard deviation (SD) are shown for each distribution. Only sites with valid coverage in both conditions are depicted.
(EPS)

S6 Fig Comparison of m^6^A modification levels across different conditions and non-DRACH and DRACH motifs. (A-H) Histograms showing the distribution of m^6^A modification percentage between knockdown (KD) and control (CTRL) conditions in all reads (A-B) and downsampled reads (C-H) for the indicated datasets and motifs. Only sites with valid coverage in both conditions are depicted. Y-axes are log-scaled to visualize the full range of the distribution.
(EPS)

S7 Fig Comparison of modified sites in coding regions across different conditions and non-DRACH and DRACH motifs. (A-L) Histograms showing the frequency of genes containing different numbers of unique modified coding sequence (CDS) sites for all reads (A-C), and downsampled reads (D-L) in the indicated datasets in non-DRACH and DRACH/GGACU motifs. Y-axes are log-scaled to visualize the full range of the distribution. The number of genes, sites, and mean and median differences are shown for each distribution.
(EPS)

S8 Fig Gene expression levels, m^6^A modification levels, and poly(A) tails. (A, B) qPCR-based analysis of m^6^A-modified transcripts with 20%, 50%, and 90% methylation levels, demonstrating changes in expression levels following knockdown (KD) of METTL3/14 compared to control (CTRL) in (A), and of m^6^A-modified transcripts displaying an increase in m^6^A modification following knockdown of METTL3 in (B) across three technical replicates. Normalized Ct values (ΔCt) to GAPDH expression are displayed as mean ± standard deviation of the mean. *p < 0.05, **p < 0.01, ***p < 0.001, and ****p < 0.0001; two-way ANOVA with Dunnett’s multiple comparisons test performed on normalized Ct values. (C) Percentage of the poly(A) tail-containing reads, binned by CTRL poly(A) tail length quartiles. (D) Bar plots of poly(A) lengths in each sample among transcripts with different m^6^A modification levels measured by publicly available GLORI in HeLa cells. m^6^A groups were characterized as transcripts with no m^6^A sites detected (No m^6^A), transcripts with m^6^A sites with a mean modification ratio of ≤ 0.5 (Low m^6^A), and transcripts with a mean m^6^A modification ratio of ≥ 0.5 (High m^6^A). (E-H) GO analysis of genes categorized based on poly(A) tail and m^6^A levels in (E, F), and gene expression and poly(A) tail in (G, H). m^6^A, N^6^-methyladensoine.
(EPS)

S9 Fig m^6^A sites mapping along selected genes in control and METTL3/14 knockdown. The m^6^A sites are shown as dots for each of the indicated datasets, with the color of each dot corresponding to the modification ratio of the corresponding m^6^A site in each dataset. CTRL, control; KD, knockdown; chr, chromosome; UTR, untranslated region; and CDS, coding region.
(EPS)

S10 Fig Poly(A) tail length distribution for selected genes following METTL3/14 knockdown. (A-D) Reads are binned by tail length quartiles; 1–60 nt, 61–107 nt, 108–161 nt, and ≥ 162 nucleotides. Stacked bar charts show the length distribution of poly(A) tails for the indicated genes with each category of length corresponding to a quartile of tail lengths in control (CTRL). Genes are grouped according to their corresponding transcripts’ baseline m^6^A modification ratios (20%, 50%, 90%) and their modification levels in METTL knockdown (KD); decreased m^6^A levels in METTL3/14 (A-C), and increased m^6^A modification in METTL3 (D).
(EPS)

S1 Table Primers used for qPCR amplification and SELECT assays. Phos, phosphate group; F, forward; R, reverse; (*) unmodified adenosine.
(XLSX)

S2 Table TERA-Seq libraries from the RNA002 and RNA004 chemistries. (*), two merged biological libraries; nt, nucleotides; CTRL, control; KD, knockdown.
(XLSX)

S3 Table Transcriptome-wide levels of METTL3 and METTL14 in RNA002/RNA004 libraries. CPM, count per million; CTRL, control; KD, knockdown.
(XLSX)

S4 Table Number of Dorado modifications called before and after false-positive correction in control dataset. m^6^A, N^6^-methyladenosine; m^5^C, 5-methylcytosine; Ψ, pseudouridine; IVT, *in vitro* transcribed. (XLSX)

S5 Table Number of Dorado modifications called before and after false-positive correction in METTL3 KD dataset. m^6^A, N^6^-methyladenosine; m^5^C, 5-methylcytosine; Ψ, pseudouridine; IVT, *in vitro* transcribed. (XLSX)

S6 Table Number of Dorado modifications called before and after false-positive correction in METTL14 KD dataset. m^6^A, N^6^-methyladenosine; m^5^C, 5-methylcytosine; Ψ, pseudouridine; IVT, *in vitro* transcribed. (XLSX)

S7 Table Overlap statistics for comparison across conditions between m6Anet and Dorado calling models. CTRL, control; KD, knockdown.
(XLSX)

S8 Table Overlap statistics for comparison across subsampled library read counts. CTRL, control; KD, knockdown; Mods, modifications.
(XLSX)

S9 Table Primers used for qPCR amplification of METTL3/14 targets. F, forward; R, reverse.
(XLSX)

S10 Table Overlapping m6A and poly(A) length changes in METTL3 KD vs. CTRL compared to METTL14 KD vs. CTRL. KD, knockdown; m^6^A, N^6^-methyladenosine.
(XLSX)
